# Modified *Sijunzi* decoction in the treatment of ulcerative colitis in the remission phase: study protocol for a series of *N*-of-1 double-blind, randomised controlled trials

**DOI:** 10.1186/s13063-020-04315-0

**Published:** 2020-05-12

**Authors:** Yi-ming Chen, Jie-min Deng, Yi Wen, Bin Chen, Jiang-tao Hou, Bin Peng, Shi-jing Zhang, Hong Mi, Qi-long Jiang, Xia-lin Wu, Feng-bin Liu, Xin-lin Chen

**Affiliations:** 1 School of Basic Medical Science, Guangzhou University of Chinese Medicine, Guangzhou, China; 2 The First Clinical College, Guangzhou University of Chinese Medicine, Guangzhou, China; 3 The First Affiliated Hospital, Guangzhou University of Chinese Medicine, Guangzhou, China

**Keywords:** Sijunzi decoction, Mesalazine, Ulcerative colitis, *N*-of-1 trial, Randomised controlled trial

## Abstract

**Background:**

Modified *Sijunzi* decoction (SJZD) has been used to treat ulcerative colitis (UC) in remission. However, more rigorous clinical trials are necessary to evaluate its effectiveness. Therefore, a series of single-case randomised controlled trials (*N*-of-1 trials) is proposed to compare the efficacy of modified SJZD with mesalazine for treating UC in remission.

**Methods:**

This is a single-site, hospital-based, double-blind *N*-of-1 trial for 10 single subjects. Three cycles of *N*-of-1 trials are planned. There are two treatment periods in each cycle. Modified SJZD combined with mesalazine placebo or mesalazine combined with modified SJZD placebo will be randomised during each 8-week treatment period. There is no washout period in the study. Subjects will be selected by the researcher strictly in accordance with the inclusion and exclusion criteria.

**Discussion:**

Paired *t* tests and mixed-effect models will be used to analyse the visual analogue scale (VAS) for clinical symptoms and the quality of life questionnaire responses. The findings will be interpreted with caution. We anticipate that the results will show that modified SJZD is effective for patients with UC in remission.

**Trial registration:**

Chinese Clinical Trial Register, ID: ChiCTR1900024086. Registered on 24 June 2019.

## Background

Ulcerative colitis (UC) is a chronic, relapsing inflammatory bowel disease that is localised to the colonic mucosa and has an unknown aetiology. Its main clinical manifestations include bloody stools, diarrhoea, and abdominal pain [1]. The incidence of UC ranges from 0.42 to 4.6 per 100,000 people-years in Eastern Asia, while the incidence in Western countries remains high as prevalence surpasses 0.3% [2]. It is anticipated that the incidence difference between Eastern and Western countries will decrease [3–5]. UC is a complex and costly disease affecting the productivity and quality of life (QOL) of patients, which incurs a significant economic and mental burden.

The long-term pharmacological treatment for UC in remission includes 5-aminosalicylic acid (5-ASA), corticosteroids, and immunosuppressant agents. 5-ASA, also known as mesalazine, is generally used as a first-line treatment for patients with mild to severe UC [6]. Mesalazine is recommended by the Asia Pacific Association of Gastroenterology (APAGE) for the treatment of UC in remission [6]. However, a subpopulation of patients is intolerant or refractory to mesalazine or unable to tolerate the adverse effects of this therapy, resulting in increased susceptibility to infection and the production of low responders [7]. Traditional Chinese medicine (TCM) has been used to treat UC as an adjunctive or alternative therapy, and has had a positive impact on improving patients’ clinical symptoms and QOL through the relief of abdominal pain, diarrhoea, and inflammation [8–10].

*Sijunzi* decoction (SJZD) is the basic formulation of TCM for strengthening the spleen and replenishing *qi*. Modified SJZD is based on SJZD and adds another Chinese medicine to treat the specific symptoms of UC [11]. SJZD or modified SJZD has been widely used to treat patients with UC in remission [12–15]. Experimental research has identified numerous pharmacological effects of SJZD, such as promoting digestion and absorption, regulating gastrointestinal motility, creating resistance to gastrointestinal mucosal injury and enhancing intestinal mucosal immunity [16–19]. There have been several clinical observations using modified SJZD independently to treat UC in China that have reported that modified SJZD was effective [20–23]. However, because of the risks of bias reported in those clinical studies, further clinical evidence and rigorous randomised controlled trials (RCTs) are needed to confirm the results.

### Rationale for using *N*-of-1 trials

A series of *N*-of-1 trials was chosen for the following reasons:

1. UC is characterised by repeated relapse and remission [1]. A realistic treatment approach is to maintain remission for a long period after remission induction [1]. Patients need personalised treatment to alleviate prolonged suffering. UC meets the required conditions of *N*-of-1 trials
2. RCTs are not suitable for all diseases and treatments. *N*-of-1 trials can be considered for nonprogressive chronic disease [24, 25]. The *N*-of-1 trial is designed for a single patient or a series of individual patients. Multiple randomised crossover trials are alternated between the intervention period and the control period [26, 27]. *N*-of-1 trials have been placed at the pinnacle of the evidence hierarchy by the Oxford Centre for Evidence-Based Medicine, with the systematic review of RCTs being in second place [28]. Compared with RCTs or crossover trials, *N*-of-1 trials have higher power and require smaller sample sizes [29]
3. *N*-of-1 trials meet the characteristics of syndrome differentiation and the individual medical interventions in TCM. Different interventions can be set up for each patient, and this design can provide a more flexible clinical trial design for TCM

### Objectives

This study aims to compare the efficacy of modified SJZD with mesalazine to treat patients with UC in remission using a series of *N*-of-1 trials.

## Methods

### Trial design

Three cycles of *N*-of-1 trials were chosen for the following reasons: (1) *N*-of-1 trials generally involve three or more cycles. Three cycles are the most commonly planned number of treatment cycles [30] and (2) for *N*-of-1 trials, the number of cycles is a variable in the sample size calculation [29]. Different cycles result in different sample sizes. Longer cycles will result in a higher dropout rate.

The *N*-of-1 trials will consist of three cycles, each cycle comprising 8 weeks of an intervention period and 8 weeks of a control period, in a random order. There is no washout period in this study. The protocol conforms to the Standard Protocol Items: CONSORT extension for reporting *N*-of-1 trials (CENT) 2015 [26]. Table 1 shows the schedule of enrolment, interventions, and assessments.

**Table 1:** Schedule of enrolment, interventions, and assessments

Time point		Enrolment	Allocation	Cycle1		Cycle2		Cycle3		Close-out
				Weeks1–8	Weeks9–16	Weeks17–24	Weeks25–32	Weeks33–40	Weeks41–48	
Enrolment	Eligibility screen	**X**								
	Informed consent	**X**								
	Allocation		**X**							
Intervention	Modified *Sijunzi* decoction combined with mesalazine placebo or mesalazine combined with modified *Sijunzi* decoction placebo			**X**	**X**	**X**	**X**	**X**	**X**	
Assessment	Laboratory tests	**X**		**X**	**X**	**X**	**X**	**X**	**X**	**X**
	Vital signs	**X**		**X**	**X**	**X**	**X**	**X**	**X**	**X**
	Adverse events			**X**	**X**	**X**	**X**	**X**	**X**	
	VAS (abdominal pain, diarrhoea, bloody stool, mucus stool), stool frequency, SIBDQ, RFIPC, SHS, and TCM syndrome scale	**X**		**X**	**X**	**X**	**X**	**X**	**X**	

*RFIPC* Rating Form of Inflammatory Bowel Disease Patient Concerns*, SHS* Short Health Scale*, SIBDQ* Short Inflammatory disease Patient Concerns, *TCM* traditional Chinese medicine, *VAS* visual analogue scale

### Participants

All UC patients will be enrolled from the First Affiliated Hospital of Guangzhou University of Chinese Medicine (GZUCM). Ethical approval was obtained from the Ethics Committee of The First Affiliated Hospital of GZUCM.

The patients will be included if they meet the following inclusion criteria:

1. Patients diagnosed with UC in the remission phase (determined by a UC Disease Activity Index score of 0–2 and a bloody stool score of 0) according to ‘The Asia-Pacific consensus on ulcerative colitis’ promulgated by APAGE on Inflammatory Bowel Disease in 2010 [6]
2. Patients diagnosed with dampness, stagnancy, and spleen deficiency syndrome according to ‘The experts of TCM consensus on ulcerative colitis (2017)’ promulgated by the Spleen and Stomach Diseases Branch of the Chinese Society of TCM [11]. The symptoms included abdominal distension and pain, relatively high frequency of stool, production of thin faecal matter, a red and white, sticky, jelly-like stool or indigested food in the stool, pale complexion, mental fatigue, pale tongue with greasy coating (moss), and weak pulse
3. Patients aged between 18 and 75 years
4. Patients who agree to voluntary participation in this study, sign the informed consent form, and have good compliance

The patients will be excluded according to the following criteria:

1. Patients with active UC, severe UC, or the acute fulminating type of UC
2. Patients undergoing treatment with corticosteroids (orally administered, enemas, suppository, or injectable solution)
3. Patients treated with blood-cell apheresis
4. Patients who were administered anti-tumour necrosis factor-a antibody within 90 days before starting the study drug
5. Patients with a history of hypersensitivity to mesalazine and salicylic acid
6. Patients with serious cardiovascular disease, haemodyscrasia, or lung disease or with a history of these conditions
7. Patients with liver disease or kidney disease
8. Patients with a malignant tumour as a complication;
9. Pregnant women or those who are breastfeeding

Patients will be withdrawn from the trials if they meet the following criteria:

1. Patients whose symptoms worsen and whose Mayo Disease Activity Index score (Mayo scores) increase by ≥ 30% during the treatment, compared with the baseline
2. Patients with serious adverse reactions during treatment, or patients who voluntarily quit or are found to be ineligible for the study by the investigators
3. Patients whose total medication that is not within 80 to 120% of the total required amount of medication after the trial
4. Personal patient request for withdrawal due to health considerations or a withdrawal request by the investigator

### Interventions

Patients in the intervention period will be treated with 300 ml modified SJZD granules and 1 g mesalazine placebo three times each day, while patients in the control period will be given 1 g mesalazine three times and 300 ml modified SJZD granules placebo each day. Each period lasts for 8 weeks.

Modified SJZD is a combination of *Radix pseudostellariae* (*Taizishen*), *Atractylodes macrocephala* (*Baizhu*), Poria (*Fuling*), *Portulaca oleracea* L (*Machijian*), *Rhizoma cimicifugae* (*Shengma*), *Citrus aurantium* L (*Zhike*), and *Agastache rugosa* (*Huoxiang*). If the symptom of bloody purulent stool is severe, *Polygonum chinense* L (*Huotanmu*) and *Crinis carbonisatus* (*Xueyutan)* will be added. If the symptoms of tenesmus are severe, *Verbena officinalis* L (*Mabiancao*), *Paris yunnanensis* Franch (*Chonglou*), and *Indigo naturalis* (*Qingdai*) will be added. All ingredients will be manufactured as a Chinese herbal granule by Guangdong Yifang Pharmaceutical Co. Ltd. Mesalazine enteric-coated tablets (Dr. Falk Pharma GmbH, Freiburg, Germany) will be used in the study. Guangdong Yifang Pharmaceutical Co. Ltd. makes both of the placebos, which have an identical shape, colour, and weight as the active drugs.

### Outcomes

The primary outcomes for this trial are the changes in the visual analogue scale (VAS) for diarrhoea, abdominal pain, mucus in the stool, and bloody purulent stool. The secondary outcomes will include the TCM syndrome scale, the Short Inflammatory Bowel Disease Questionnaire (SIBDQ), the Rating Form of Inflammatory Bowel Disease Patient Concerns (RFIPC), and the Short Health Scale (SHS). Efficacy indicators for each subject will be observed and recorded before the study and at the end of each period (including week 8, week 16, week 24, week 32, week 40, and week 48).

Patients will be asked to rate their diarrhoea, abdominal pain, mucus in the stool, and bloody purulent stool severity by placing a vertical mark on a 10-mm horizontal VAS. The left and right extremes of the VAS are labelled ‘the lightest’ and ‘the most serious’, respectively.

The (TCM) Traditional Chinese medicine syndrome scale is an efficacy evaluation of six items measuring dampness, stagnancy due to spleen deficiency syndrome: diarrhoea, bloody purulent stool, anorexia, abdominal distension, limb burnout, and disinclination to talk due to mental weariness. Each item is rated from 0 to 3. The higher the scale, the more serious is the dampness stagnancy [11].

The SIBDQ uses 10 items derived from the inflammatory bowel disease questionnaire, which explain 90% of the variance in the SIBDQ for UC patients. The total score ranges from 10 (worst health) to 70 (best health). This gauge of subjective health status or quality of life in patients can be administered and scored quickly and easily [31].

The RFIPC is a self-administered form containing 25 items of concern related to inflammatory bowel disease. Each item is rated from 0 to 100 on a VAS form. The average score of all 25 items is the ‘sum score’. A higher score indicates a higher level of patient anxiety [32].

The SHS is a disease-specific questionnaire consisting of four items measuring four dimensions of health: bowel symptom severity, social functioning in daily life, disease-related worry, and perceptions of general well-being. Item responses are indicated on a 100-mm VAS from 0 to 100. The higher the scale, the more serious are the health concerns [33].

### Safety assessment

The following safety indicators will be measured before the study and at the end of each period (including week 8, week 16, week 24, week 32, week 40, and week 48):

1. Laboratory tests, including routine blood tests (white blood cell count, total number of neutrophils, total number of lymphocytes, total number of monocytes, total number of eosinophils, total basophils, percentage of neutrophils, lymphocyte percentage, percentage of monocytes, eosinophil percentage, basophil percentage, total number of red cells, haemoglobin volume, erythrocyte specific volume, mean erythrocyte volume, mean erythrocyte haemoglobin content, mean erythrocyte haemoglobin concentration, erythrocyte distribution width, total platelet count, mean platelet volume, platelet distribution width, and specific platelet volume), routine urine tests (urine specific gravity, uric acid alkalinity, urine leukocyte lipase, nitrite, urine protein, urine glucose, urine ketone body, uric bravery former, uric bilirubin, occult blood, white blood cell count, red blood cell count, epithelial cells, crystallisation, small-round-cell lipids, trichomonad fungus, tube type, and amorphous salt crystallisation), liver function tests (alanine transaminase, glutamyl transpeptidase), kidney function tests (serum creatinine), and blood lipids
2. Vital signs including blood pressure, respiration rate, heart rate, body temperature, 12-lead electrocardiogram, and physical examination
3. All adverse events occurring during the study, including toxicities and side effects, such as rash, gastrointestinal discomfort (abdominal pain, nausea, and vomiting), drug allergies, liver damage, and renal failure, will be reported and recorded in the case report form (CRF) in detail

### Sample size

Based on published data [20], whose primary outcome was the VAS score of mucus in the stool, the standard deviation of self-efficacy was 0.8, and the difference value of efficacy between the two groups was 0.7. We assume that there is no difference in efficacy between each patient. Assuming a two-sided significance level of 0.05 and power of 80% (*α* = 0.05, *β* = 0.2), the sample size is calculated as 8 (fixed model) [29]. Considering a 20% loss to follow-up, the sample size was 10 cases.

### Randomisation, treatment allocation, and blinding

The sequence of the test period was randomly assigned within each of three pairs of periods using an SAS random programme. There are two treatment periods in each cycle, the intervention period (A) and the control period (B). Only AB and BA are allowed in each cycle. The random programme will be created by statisticians from the School of Basic Medical Science of GZUCM. According to this, the test drugs will be randomly hand out by the researcher who will not participate in the trial. The prepared research drugs will be delivered, stored, and distributed in the First Affiliated Hospital of GZUCM. Patients and investigators will be blinded to all randomisation and packaging procedures until completion of the trial.

The modified SJZD (or mesalazine) placebo tablets will closely resemble the appearance, odour, and taste of the active modified SJZD granules (mesalazine). Placebos will be packaged in the same way as the active tablets to minimise the risk of breaking the blind.

### Statistical methods

All data will be recorded and collected on the CRFs by the researchers. The data will be input using EpiData 3.0 (EpiData Association, Odense, Denmark). To ensure the accuracy and integrity of the data, double data entry and proofreading will be performed by two independent researchers.

R software (version 3.6.2) will be utilised for the statistical analysis. A *P* value ≤ 0.05 will be considered statistically significant. For the quantitative indicators, the mean and standard deviation or the median and interquartile range will be calculated. Descriptive statistics will be used to summarise the demographic characteristics of the participants, including age, sex, and extent of UC.

For the individual *N*-of-1 trials, paired *t* tests or Wilcoxon signed-rank tests will be used to compare the efficacy (including the VAS for diarrhoea, abdominal pain, mucus in the stool, and bloody purulent stool, as well as all the QOL scores) of modified SJZD with that of mesalazine. For individual patient reports, the VAS for diarrhoea, abdominal pain, mucus in the stool, and bloody purulent stool, as well as all the QOL scores and safety indicators reported, will be described, as will whether the efficacy is within the range of significant improvement.

To synthesise the data from the *N*-of-1 trials from all patients (including the VAS for diarrhoea, abdominal pain, mucus in the stool, and bloody purulent stool, as well as all the QOL scores), a paired *t* test was used for the analysis. Considering the correlated data structure, we also used a mixed model. The equation for the mixed model is as follows [34]:

1 $$ {\displaystyle \begin{array}{l}{Y}_i={X}_i{\theta}_i+{\varepsilon}_i\kern0.5em \mathrm{with}\kern0.5em {\varepsilon}_i\sim N\left(0,{\Sigma}_i\right)\\ {}{\theta}_i\sim N\left(\theta, D\right)\end{array}} $$

where *Y*_*i*_ = (*Y*_*i1*_, *Y*_*i2*_, …, *Y*_*ij*_) denotes the *i*-th (*i* = 1, 2, …, *n*) subject’s response value for the *j*-th (*j* = 1, 2, …6) period; *θ*_*i*_ follows a multivariate normal distribution with mean *θ* and a covariance matrix *D*. *θ*_*i*_ contains treatment effect (μ_i_) between mesalazine and modified SJZD. When *μ*_*i*_ is same for each patient (*μ*_*i*_ *= μ*), it is a fixed effect. If *μ*_*i*_ varies across different patients, it is a random effect. Some covariance structures for the random error are assumed, including: (1) compound symmetry (common variance across patients with uncorrelated and identically distributed errors), (2) first-order autoregressive, and (3) unstructured: which may be overparameterised. In these models, other factors (carryover effects and period effects) are also considered. The mixed models are fitted using the ‘lme4’ package in R software (version 3.6.2). Different models are compared using the Bayesian information criterion (BIC) [35]. Finally, we prefer the model with the lowest BIC.

## Discussion

A sufficient washout period is necessary for *N*-of-1 trials because the latter intervention may be affected by the carryover effects of the previous intervention. However, the patient does not receive effective treatment during washout periods. Therefore, including a washout period would be inappropriate for UC patients. For patients with UC in the remission phase, insufficient treatment leads to acute UC or aggravation of UC [36, 37]. Therefore, there are no washout periods in our study.

Although there are no washout periods in this study, we considered that the carryover effects of modified SJZD have little effect in practice. The reasons are as follows: (1) the main active ingredients of SJZD are glycyrrhizic acid, glycyrrhetinic acid, pachymic acid, atractylodes lactone III, ginsenoside Re, and ginsenoside Rb_1_, and a pharmacokinetic study showed that their mean half-life is no more than 12 h [38]. For the other active ingredients of modified SJZD, further studies reported that their half-life is no more than 24 h [39–45] and (2) even if there are carryover effects for modified SJZD, a mixed-effect model will be used to analyse the data from the *N*-of-1 trials. The mixed-effects model can estimate treatment effects accurately while controlling for carryover effects; it separates carryover effects from treatment effects in a series of *N*-of-1 trials [34].

The Mayo score is a general indicator used to assess the severity of active UC [6]. If the procedure for calculating the Mayo score is followed properly, the patients with UC would undergo enteroscopy after each period (one per month in the trial). This would be too frequent for patients to accept. Therefore, the Mayo score will not be used as the primary outcome. Diarrhoea, abdominal pain, and mucus in the stool are the most common symptoms for patients with UC. Bloody purulent stool was reported to have a close relationship with clinical relapse [46, 47]. For these reasons, the VAS scores for the main clinical symptoms (diarrhoea, abdominal pain, mucus in the stool, and bloody purulent stool) were chosen as the primary outcomes.

Our findings will be interpreted with caution. We anticipate that the results will show that modified SJZD is effective for patients with UC in remission.

### Trial status

The recruitment will begin on 1 March 2020, and we expect this study to be completed by 30 February 2022.

## Data Availability

The data used to support the findings of this study will be available from the Chinese Clinical Trial Register (ChiCTR1900024086) within 6 months after the trial is complete.

## Abbreviations

UC: Ulcerative colitis
QOL: Quality of life
TCM: Traditional Chinese medicine
SJZD: *Sijunzi* decoction
*N*-of-1: Single-case randomised controlled
RCT: Randomised controlled trial
GZUCM: Guangzhou University of Chinese Medicine
CENT: CONSORT extension for reporting *N*-of-1 trials
VAS: Visual analogue scale
SIBDQ: Short Inflammatory Bowel Disease Questionnaire
RFIPC: Rating Form of Inflammatory Bowel Disease Patient Concerns
SHS: Short Health Scale
APAGE: Asia Pacific Association of Gastroenterology
CRF: Case report form
